# Comprehensive off-target analysis of dCas9-SAM-mediated HIV reactivation via long noncoding RNA and mRNA profiling

**DOI:** 10.1186/s12920-018-0394-2

**Published:** 2018-09-10

**Authors:** Yonggang Zhang, Gustavo Arango, Fang Li, Xiao Xiao, Raj Putatunda, Jun Yu, Xiao-Feng Yang, Hong Wang, Layne T. Watson, Liqing Zhang, Wenhui Hu

**Affiliations:** 1 Center for Metabolic Disease Research, Temple University Lewis Katz School of Medicine, 3500 N Broad Street, Philadelphia, PA 19140 USA; 2 Center for Stem Cell Research and Application, Institute of Blood Transfusion, Chinese Academy of Medical Sciences & Peking Union Medical College (CAMS & PUMC), Chengdu, 610052 China; 3 Department of Computer Science, Virginia Tech, Blacksburg, VA 24060 USA; 4 Department of Mathematics, Department of Aerospace and Ocean Engineering, Virginia Tech, Blacksburg, VA 24060 USA; 5 Department of Pathology and Laboratory Medicine, Temple University Lewis Katz School of Medicine, 3500 N Broad Street, Philadelphia, PA 19140 USA

**Keywords:** Genome editing, CRISPR, Off-target, RNA sequencing, Transcriptome, HIV, Latency, Shock and kill

## Abstract

**Background:**

CRISPR/CAS9 (epi)genome editing revolutionized the field of gene and cell therapy. Our previous study demonstrated that a rapid and robust reactivation of the HIV latent reservoir by a catalytically-deficient Cas9 (dCas9)-synergistic activation mediator (SAM) via HIV long terminal repeat (LTR)-specific MS2-mediated single guide RNAs (msgRNAs) directly induces cellular suicide without additional immunotherapy. However, potential off-target effect remains a concern for any clinical application of Cas9 genome editing and dCas9 epigenome editing. After dCas9 treatment, potential off-target responses have been analyzed through different strategies such as mRNA sequence analysis, and functional screening. In this study, a comprehensive analysis of the host transcriptome including mRNA, lncRNA, and alternative splicing was performed using human cell lines expressing dCas9-SAM and HIV-targeting msgRNAs.

**Results:**

The control scrambled msgRNA (LTR_Zero), and two LTR-specific msgRNAs (LTR_L and LTR_O) groups show very similar expression profiles of the whole transcriptome. Among 839 identified lncRNAs, none exhibited significantly different expression in LTR_L vs. LTR_Zero group. In LTR_O group, only TERC and scaRNA2 lncRNAs were significantly decreased. Among 142,791 mRNAs, four genes were differentially expressed in LTR_L vs. LTR_Zero group. There were 21 genes significantly downregulated in LTR_O vs. either LTR_Zero or LTR_L group and one third of them are histone related. The distributions of different types of alternative splicing were very similar either within or between groups. There were no apparent changes in all the lncRNA and mRNA transcripts between the LTR_L and LTR_Zero groups.

**Conclusion:**

This is an extremely comprehensive study demonstrating the rare off-target effects of the HIV-specific dCas9-SAM system in human cells. This finding is encouraging for the safe application of dCas9-SAM technology to induce target-specific reactivation of latent HIV for an effective “shock-and-kill” strategy.

**Electronic supplementary material:**

The online version of this article (10.1186/s12920-018-0394-2) contains supplementary material, which is available to authorized users.

## Background

Recently, CRISPR/Cas9 genome editing technology has been rapidly developed and attracted extensive attention in biomedical research, with preclinical examples and potential clinical trials in genetic diseases, cancer biology, and infectious diseases [1–7]. Simultaneously, the catalytically-deficient Cas9 (dCas9) epigenome editing technology has emerged as a novel platform for the manipulation of cellular or viral gene regulation by incorporating monoplex or multiplex transcriptional activators or repressors [8–19]. Cas9-mediated genome editing technology has been utilized to excise the HIV-1 provirus via HIV-specific multiplex single guide RNAs (sgRNAs) in cultured HIV latent cell lines [20–22], primary T cells [22, 23], and HIV transgenic rodents [24, 25]. The dCas9 epigenome editing technology [8–11, 19] is also used to reactivate the latent HIV-1 provirus using HIV long terminal repeat (LTR)-specific sgRNAs [26–29]. A rapid and robust reactivation of the HIV latent reservoir by dCas9-synergistic activation mediator (SAM) via MS2-mediated sgRNAs (msgRNAs) [30] directly induces cellular suicide without additional immunotherapy [31], which might be a novel, practical, and specific method for the “shock and kill” strategy to cure HIV/AIDS. The dCas9-SAM approach also induces specific activation of endogenous viral restriction factors that affect virus replication [32].

In addition to transcriptional activation, the dCas9 property is also extensively repurposed for transcriptional repression and DNA (de)methylation [12, 33–35]. These epigenome-editing approaches can alter the epigenetic code of the target region, and thus offer a durable manipulation of many genes important in infectious diseases, cancer, and chronic noninfectious diseases [12, 36]. Modification of an individual chromatin mark may suppress target gene expression in most cases [36]. However, permanent silencing of target genes in all cell types may require a combination of several epigenetic effectors [12].

Potential off-target effect remains a critical concern for any clinical application of this technology. Several promising strategies have been developed to mitigate any potential off-target responses, such as the sgRNA design optimization [37–42], transcriptome analysis [28, 30], and functional screening after dCas9 treatment [43]. For the parent Cas9 genome editing system, increasing experimental data suggests that the genome editing is highly specific [20, 44–48]. Newly developed unbiased profiling techniques further validate the high specificity of this Cas9/sgRNA technology [49–54]. In vivo off-target effects are expected to be low due to epigenetic protection [55, 56]. Specifically for dCas9 technology, the frequency of off-target binding to essential (functional) exons would also be very low [57]. Further mRNA-seq analysis confirmed the specificity of this dCas9-SAM technology [28, 30].

Our previous studies analyzed the exogenous viral DNA against the host genome for the best scores of efficiency and specificity [20, 21, 31]. In TZM-bI cells expressing the HIV LTR-driven luciferase reporter without the viral genome itself [58], the dCas9-SAM technology with HIV LTR-specific msgRNAs induced potent reactivation of the HIV reporter, but did not influence the cell growth/proliferation [31], supporting the absence of off-target effects by the dCas9-SAM technology [27, 28, 59]. The aim of this study is to further explore the dCas9-SAM-related potential off-target effects by generating deep sequence coverage of the entire transcriptome, comprehensively analyzing mRNAs, lncRNAs, alternative splicing, genetic mutations including single-nucleotide polymorphisms (SNPs) and indels (insertions and deletions) in TZM-bI cells stably expressing dCas9-SAM and HIV-specific msgRNAs. These analyses are important for safety considerations during the potential clinical application of dCas9 epigenome editing technology [60].

## Methods

### Experimental design and RNA sample preparation

The HeLa cell-derived TZM-bl cell line stably expressing higher levels of CD4 and CCR5 was obtained from Dr. John C. Kappes through the NIH AIDS Reagent Program, Division of AIDS, NIAID, NIH. It was generated by introducing separate integrated copies of the luciferase and ß-galactosidase genes under control of the HIV-1 LTR promoter. To establish the dCas9-SAM stable expression cell line (designated TZMb-6465 cell line), TZM-bI cells were transduced with pMSCV-dCas9-BFP (puromycin) retroviral vector (Addgene, plasmid #46912) [10], and Lenti-MS2-p65-HSF1 (hygromycin) lentiviral vector (Addgene, plasmid #61426) [30]. After 2 days, cells were subcultured and selected with puromycin (2 μg/ml) and hygromycin (200 μg/ml). After 2 weeks of selection culture, the TZMb-6465 cells were transduced with msgRNA-expressing empty control lentiviral vector (Addgene, Plasmid #61427) [30], HIV-1 LTR_L msgRNA-expressing lentivirus or LTR_O msgRNA-expressing lentivirus. Six samples were prepared: two replicates for the LTR_L editing (LTR_L1 and LTR_L2), two replicates for the LTRO editing (LTR_O1 and LTR_O2), and two replicates for control (LTR_Zer1 and LTR_Zer2). After four days, cells were subjected to total RNA extraction using the Direct-Zol RNA MiniPrep Kit (Genesee Scientific, Catalog number: 11–330). The 4-day post-infection time point was based on the sufficient msgRNA expression and potent LTR-target reactivation [31] while minimizing the possible confounding factor resulting from the indirect downstream effects of any potential off-targets, if they existed. The RNAs were preserved with RNAstable LD (Sigma, Catalog number: 53201–013) and shipped to Novogene Bioinformatics Institute (https://en.novogene.com/) for total RNA sequencing and bioinformatics analysis. The RNA integrity was verified by 1% agarose gel electrophoresis and Agilent 2100. The RNA purity was checked using a NanoPhotometer® spectrophotometer (IMPLEN, CA, USA) and the DNA concentration was measured using Qubit® DNA Assay Kit in Qubit® 2.0 Fluorometer (Life Technologies, CA, USA).

### Library construction and sequencing

The RNA quality control (QC) was done using Trimmomatic with default settings, and this step discarded less than 3% of the RNA reads, and the results were shown in Additional file 1: Table S1. After RNA QC, rRNAs were removed by using the Epicentre Ribo-Zero™ Kit. The purified RNAs were first fragmented randomly into short fragments of 150~ 200 bp by addition of a fragmentation buffer, then cDNA synthesis was performed using random hexamers. After the first strand was synthesized, a custom second strand synthesis buffer (Illumina), dNTPs (dUTP, dATP, dGTP and dCTP) and DNA polymerase I were added to synthesize the second strand, then followed by purification by AMPure XP beads, terminal repair, polyadenylation, sequencing adapter ligation, size selection, and degradation of the second strand U-contained cDNA by the USER enzyme. The strand-specific cDNA library was generated after the final PCR enrichment. The concentration of the library was first quantified by Qubit2.0, then diluted to 1 ng/ul, and the insert size was checked by Agilent 2100 and further quantified by qPCR (library concentration > 2 nM). The libraries were then subjected to HiSeq sequencing according to the concentration and the expected data volume.

### Sequence analysis

About 60 GB of RNA sequencing data was generated for all six samples. Original RNA-Seq reads contain adapters and low quality reads that needed to be filtered out. To ensure the quality of the analysis, the sequence adapters (Oligonucleotide sequences for TruSeq™ RNA and DNA Sample Prep Kits) were removed from reads using Trimmomatic [61, 62]. Then all the trimmed reads with more than 10% ambiguous bases (N) were also removed. Finally, low quality reads with a Phred score less than 20 were removed. Additional file 1: Table S1 shows the distribution of quality reads across the L, O, and Zero samples. High quality sequences are mapped to the human genome (hg38) using TopHat2 with default parameters [63]. Overall, approximately 89% of the raw reads were mapped to the human genome (detailed mapping results are shown in Additional file 1: Table S2 and Additional file 2: Figure S1). Mapped reads were then assigned to known types of RNA using the program HTSeq with the union model (see Additional file 1: Table S3 for the distribution of mapped reads in different categories of known RNAs). To quantify the transcript abundance, the FPKM metric (number of fragments per kilobase of transcript sequence per million mapped reads) was used, which considers both the sequencing depth and the transcript length. In order to measure the reliability of the experiments through biological replicates, the Pearson correlation coefficient (*R*^2^) was calculated between all pairs of the L, O, and Zero samples. A correlation coefficient close to one indicates high similarity of gene expression profiles.

### LncRNA analysis

The detailed workflow for identifying long noncoding RNAs (lncRNAs) is shown in Additional file 2: Figure S2b. First, *cufflinks* with default parameters was used to assemble the mapped reads into transcripts and quantify transcript expression (including isoforms). Candidate long noncoding RNAs (lncRNAs) were then classified into three categories (lncRNAs, intronic lncRNAs, and antisense lncRNAs) through five filtering steps (Additional file 2: Figure S2b): (1) assembled transcripts from *cufflinks* were merged using *cuffcompare* and the merged transcripts selected if they appeared in more than one sample, (2) only transcripts with more than 200 bps and two exons were kept, (3) only those transcripts that have ≥3× coverage for at least two exons were kept, (4) transcripts with high coverage were then removed if they matched known non-lncRNAs and non-mRNA (e.g., rRNA, tRNA, snRNA, snoRNA, etc), and (5) the remaining transcripts were then removed if they matched known mRNAs. The final collection of RNAs was the candidate set of lncRNAs, intronic lncRNAs, and antisense lncRNAs. Additional file 2: Figure S3 shows the number of transcripts that were filtered in each step. After all of the five filtering steps, a total of 1615 transcripts were left in the six pooled samples.

To finally determine if a transcript is a lncRNA, four popular methods for coding potential analysis were applied: (1) CPC (Coding-Potential Calculator) [64] computes the coding potential of a transcript by matching it to the NCBI nr database using BLASTX and scoring it using a support vector machine, (2) CNCI (Coding-Non-Coding Index) distinguishes protein-coding and noncoding transcripts independent of known annotations and predicts the coding or noncoding potential based solely on the features of nucleotide triplets, (3) transcripts were translated into proteins and matched to known protein domains in Pfam [65] using HMMER3 [66] where a matched sequence is considered as having coding potential, whereas others are considered as noncoding, and (4) PhyloCSF (Phylogenetic Codon Substitution Frequency) uses genome-wide mammalian sequence alignments to calculate the coding potential of transcripts.

Functions of the lncRNAs were identified by predicting their protein-coding target genes in both a *cis-* and *trans-* manner. The *cis*-acting target prediction assumes that the function of a lncRNA is determined by its adjacent protein coding genes, and in this study, coding genes within ±100 kb of the lncRNAs were considered as *cis*-acting targets. The *trans*-acting targets were predicted based on co-expressed genes, and only those genes that had Pearson correlation coefficients greater than 0.95 with the lncRNAs were selected.

### mRNA analysis

Differentially expressed mRNAs were determined using *cuffdiff* with default parameters [67]. A network analysis of protein-protein interactions for the differentially expressed mRNAs was also conducted using the STRING database [68]. If the target genes (such as the expressed mRNAs) were not found in the database, a BLASTX search was done with an E-value of 1e-10 to identify potential protein-protein interactions.

### SNP and indel variant calling

To examine whether the dCas9-SAM technology has an effect on genetic mutations, for example, resulting in different sets of SNPs and indel mutations due to the editing, SNPs and indels were called and compared for the six samples. Specifically, SAMtools [69] and Picard [https://broadinstitute.github.io/picard/] were used to preprocess the mapped reads. SNPs and indel variants were called using the GATK2 toolkit [70]. To quantify the similarity between the sets of SNPs and indel mutations in the samples, the Jaccard Index,

$$ J=\frac{\mid {S}_1\cap {S}_2\mid }{\mid {S}_1\cup {S}_{2\mid }}, $$

where |*S*| denotes the size of set *S*, *S*_1_ is the set of SNPs/indels in one sample, and *S*_2_ is the set of SNPs/indels in another sample, is calculated for all 15 pairs of sample comparisons. The Jaccard index ranges from 0 to 1, the higher it is, the more similarity in the sets of SNPs/indels between two samples, with 0 indicating that two samples have entirely different sets of SNPs/indels and 1 indicating that two samples have the same set of SNPs/indels.

### Alternative splicing

Alternative splicing (AS) was analyzed by first classifying AS events into 12 types as illustrated in Additional file 2: Figure S4 using ASprofile [71]. Then expression levels of alternatively spliced genes were estimated using the probabilistic framework MISO (Mixture of Isoforms) [72]. MISO uses a Bayesian statistical model to give a more accurate estimate of the expression level indicated by the number of reads that covers different isoforms or exons. Differential expression of isoforms was then determined by the Bayes factor (BF) that computes the odds of differential regulation occurring. The higher the BF, the more likely the isoforms/exons are differentially regulated. A cutoff BF = 10 was applied to select the isoforms/exons that were significantly differentially regulated between conditions [72]. Five major AS events, (1) A3SS (alternative 3′ splice sites), (2) A5SS (alternative 5′ splice sites), (3) MXE (mutually exclusive exons), (4) RI (retained intron), and (5) SE (skipped exon), were analyzed.

### Statistics

All the statistical tests, including Steiger’s test, two proportion z-test, and Chi-square tests were performed in R.

## Results

### Very similar expression profiles at the whole transcriptome level among the three conditions

**Fig. 1:**
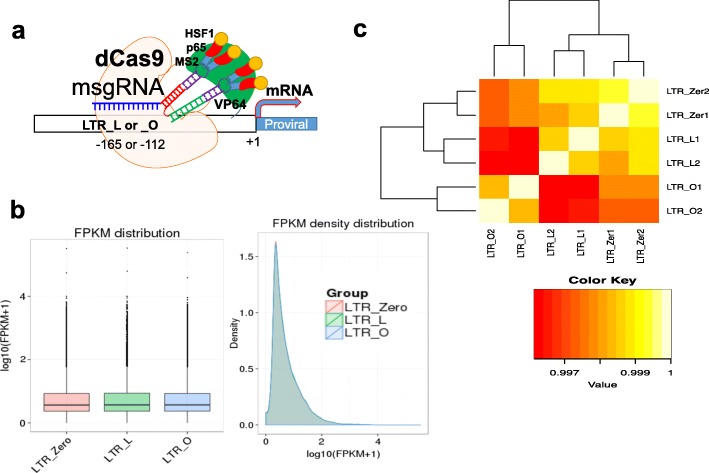
No difference in the entire RNA transcripts among the three experimental conditions. **a** Diagram showing the HIV proviral activation by the dCas9-SAM system with msgRNAs targeting LTR_L or LTR_O. **b** Box plot and density plot for the distribution of transcript expression levels measured by FPKM (averaged within replicates) of the three conditions. The plotted region of the box plot represents the maximum, upper quartile, median, lower quartile, and minimum, respectively, from top to bottom. **c** Hierarchical clustering of samples based on Pearson correlation coefficient of transcript expression levels for all the pairwise comparisons of the samples

In previous studies, 16 msgRNAs targeting the U3 region of the HIV LTR were screened for their efficiency in guiding dCas9-SAM to activate HIV promoter activity [31]. Two targeting sites, LTR_L (− 165/− 145 bp from the transcription start site) and LTR_O (− 112/− 92 bp from the transcription start site) surrounding the enhancer region (Fig. 1a), were identified for robust reactivation of HIV-1 provirus in various types of human cells [31]. These two hotspots were verified in other studies [26–29]. To determine if the dCas9-SAM system mediated by these two hotspots affects the host cells’ transcriptomes, the total RNAs from TZM-bI cells stably expressing the dCas9-SAM system plus msgRNA targeting LTR_L or LTR_O were prepared for lncRNA and mRNA sequencing. The empty msgRNA carrying scrambled target sequence was used as the control (LTR_Zero). The TZM-bI cell line was used because it harbors integrated HIV-1 LTR promoter but does not contain HIV-1 proviral DNA that may produce viral proteins leading to potential effects on the host transcriptome [58], complicating the analysis. A total of 600,451,484 raw reads were generated after read quality control and cleanup, of which 97.4% clean reads were kept for downstream analyses (see Additional file 1: Table S1 for details). The clean reads were then mapped to the human reference genome hg38 by Tophat2 [63]. More than 89% of the reads were mapped for all six samples (see Additional file 1: Table S2 for details) and distributions of the mapped reads in the genome are shown in Additional file 2: Figure S1.

The distribution of the transcript expression levels under different conditions (L, O, and Zero) was analyzed by the mean fragments per kilobase of transcript per million mapped reads (FPKM) of the two replicates for each condition (Fig. 1b). It is clear that the expression distributions of all the transcripts among the three conditions are highly similar, except for the LTR-driven reporter genes luciferase and ß-galactosidase (see Additional file 1: Table S3), which is consistent with the increased luciferase activity in the LTR-targeting groups [31]. The square of the Pearson correlation coefficient (*R*^2^) for all the transcripts among the samples and replicates was assessed, for which *R*^2^> 0.92 was considered good quality [73, 74]. Here, the correlations for all pairs of samples fell within the range of 0.9961 to 0.9993 (Fig. 1c). Samples of the same conditions (i.e., the duplicates for each condition) have significantly higher correlation coefficients than those for samples from different conditions (Steiger’s test, *p* < 0.05) [75].

Further analysis of the RNA types using HTSeq with the union model identified similar statistical analysis of the mapped reads (Table 1). Of all the reads that were mapped to RNAs, the majority of those reads, ranging from 88.74 to 89.42%, were mapped to protein coding regions, 1.71 to 2.03% to lncRNA, 3.59 to 4.76% to miscellaneous RNAs, 0.53 to 0.56% to processed transcripts, and 0.5 to 0.55% to antisense RNAs.

**Table 1:** Distribution of mapped reads in different categories of RNAs in the six samples

Sample_name	LTR_Zer1	LTR_Zer2	LTR_L1	LTR_L2	LTR_01	LTR02
3prime_overlapping_ncrna	159 (0.00%)	180 (0.00%)	160 (0.00%)	171 (0.00%)	180 (0.00%)	160 (0.00%)
IG_C_gene	0 (0.00%)	2 (0.00%)	1 (0.00%)	0 (0.00%)	3 (0.00%)	1 (0.00%)
IG_C_pseudogene	0 (0.00%)	0 (0.00%)	0 (0.00%)	0 (0.00%)	0 (0.00%)	0 (0.00%)
IG_D_gene	0 (0.00%)	0 (0.00%)	0 (0.00%)	0 (0.00%)	0 (0.00%)	0 (0.00%)
IG_J_gene	0 (0.00%)	0 (0.00%)	0 (0.00%)	0 (0.00%)	0 (0.00%)	0 (0.00%)
IG_J_pseudogene	0 (0.00%)	0 (0.00%)	0 (0.00%)	0 (0.00%)	0 (0.00%)	0 (0.00%)
IG_V_gene	4 (0.00%)	2 (0.00%)	1 (0.00%)	3 (0.00%)	1 (0.00%)	4 (0.00%)
IG_V_pseudogene	0 (0.00%)	0 (0.00%)	3 (0.00%)	1 (0.00%)	0 (0.00%)	1 (0.00%)
Mt_rRNA	1318 (0.00%)	1488 (0.00%)	1734 (0.00%)	1496 (0.00%)	1342 (0.00%)	1779 (0.01%)
Mt_tRNA	644 (0.00%)	637 (0.00%)	692 (0.00%)	784 (0.00%)	603 (0.00%)	668 (0.00%)
TEC	5415 (0.01%)	5636 (0.01%)	5036 (0.01%)	5747 (0.01%)	5685 (0.02%)	5276 (0.02%)
TR_C_gene	32 (0.00%)	21 (0.00%)	23 (0.00%)	29 (0.00%)	36 (0.00%)	26 (0.00%)
TR_D_gene	0 (0.00%)	0 (0.00%)	0 (0.00%)	0 (0.00%)	0 (0.00%)	0 (0.00%)
TR_J_gene	0 (0.00%)	0 (0.00%)	0 (0.00%)	0 (0.00%)	0 (0.00%)	0 (0.00%)
TR_J_pseudogene	0 (0.00%)	0 (0.00%)	0 (0.00%)	0 (0.00%)	0 (0.00%)	0 (0.00%)
TR_V_gene	0 (0.00%)	0 (0.00%)	0 (0.00%)	1 (0.00%)	0 (0.00%)	0 (0.00%)
TR_V_pseudogene	0 (0.00%)	0 (0.00%)	2 (0.00%)	2 (0.00%)	0 (0.00%)	2 (0.00%)
antisense	191,551 (0.52%)	204,790 (0.53%)	178,062 (0.50%)	205,243 (0.50%)	201,319 (0.54%)	189,642 (0.55%)
known_ncrna	0 (0.00%)	0 (0.00%)	1 (0.00%)	0 (0.00%)	1 (0.00%)	1 (0.00%)
lincRNA	738,731 (2.00%)	761,611 (1.97%)	706,213 (1.98%)	702,871 (1.71%)	742,207 (1.99%)	702,377 (2.03%)
miRNA	2479 (0.01%)	2557 (0.01%)	3497 (0.01%)	3299 (0.01%)	1525 (0.00%)	1430 (0.00%)
misc_RNA	1,612,667 (4.37%)	1,593,547 (4.12%)	1,627,962 (4.57%)	1,960,500 (4.76%)	1,343,420 (3.59%)	1,244,791 (3.59%)
non_coding	0 (0.00%)	0 (0.00%)	0 (0.00%)	0 (0.00%)	0 (0.00%)	0 (0.00%)
polymorphic_pseudogene	319 (0.00%)	355 (0.00%)	320 (0.00%)	369 (0.00%)	333 (0.00%)	312 (0.00%)
processed_pseudogene	10,437 (0.03%)	10,705 (0.03%)	9812 (0.03%)	11,241 (0.03%)	10,275 (0.03%)	8946 (0.03%)
processed_transcript	196,988 (0.53%)	213,355 (0.55%)	194,373 (0.55%)	229,395 (0.56%)	203,313 (0.54%)	192,191 (0.55%)
protein_coding	32,728,357	34,372,393	31,562,319	36,554,719	33,423,746	30,949,051
	(88.74%)	(88.92%)	(88.65%)	(88.74%)	(89.42%)	(89.36%)
pseudogene	147 (0.00%)	166 (0.00%)	145 (0.00%)	153 (0.00%)	172 (0.00%)	167 (0.00%)
rRNA	32 (0.00%)	44 (0.00%)	38 (0.00%)	62 (0.00%)	46 (0.00%)	44 (0.00%)
sense_intronic	1015 (0.00%)	1032 (0.00%)	1070 (0.00%)	1071 (0.00%)	1035 (0.00%)	1021 (0.00%)
sense_overlapping	14,067 (0.04%)	15,375 (0.04%)	12,765 (0.04%)	14,949 (0.04%)	15,117 (0.04%)	14,014 (0.04%)
snRNA	3417 (0.01%)	3214 (0.01%)	2915 (0.01%)	3858 (0.01%)	3149 (0.01%)	3236 (0.01%)
snoRNA	160 (0.00%)	172 (0.00%)	136 (0.00%)	149 (0.00%)	197 (0.00%)	170 (0.00%)
transcribed_processed_pseudogene	25,420 (0.07%)	26,038 (0.07%)	24,532 (0.07%)	28,196 (0.07%)	25,315 (0.07%)	23,318 (0.07%)
transcribed_unitary_pseudogene	0 (0.00%)	0 (0.00%)	0 (0.00%)	0 (0.00%)	0 (0.00%)	0 (0.00%)
transcribed_unprocessed_pseudogene	72,052 (0.20%)	77,671 (0.20%)	69,487 (0.20%)	79,027 (0.19%)	78,124 (0.21%)	73,283 (0.21%)
translated_processed_pseudogene	0 (0.00%)	0 (0.00%)	0 (0.00%)	0 (0.00%)	0 (0.00%)	0 (0.00%)
translated_unprocessed_pseudogene	1 (0.00%)	0 (0.00%)	0 (0.00%)	1 (0.00%)	0 (0.00%)	0 (0.00%)
unitary_pseudogene	7892 (0.02%)	8444 (0.02%)	7147 (0.02%)	8507 (0.02%)	8233 (0.02%)	7525 (0.02%)
unprocessed_pseudogene	12,070 (0.03%)	12,228 (0.03%)	11,432 (0.03%)	12,660 (0.03%)	12,177 (0.03%)	11,856 (0.03%)
Others	1,257,539 (3.41%)	1,342,963 (3.47%)	1,185,376 (3.33%)	1,370,001 (3.33%)	1,299,971 (3.48%)	1,201,876 (3.47%)

### Very similar expressions of lncRNAs among the three conditions

**Fig. 2:**
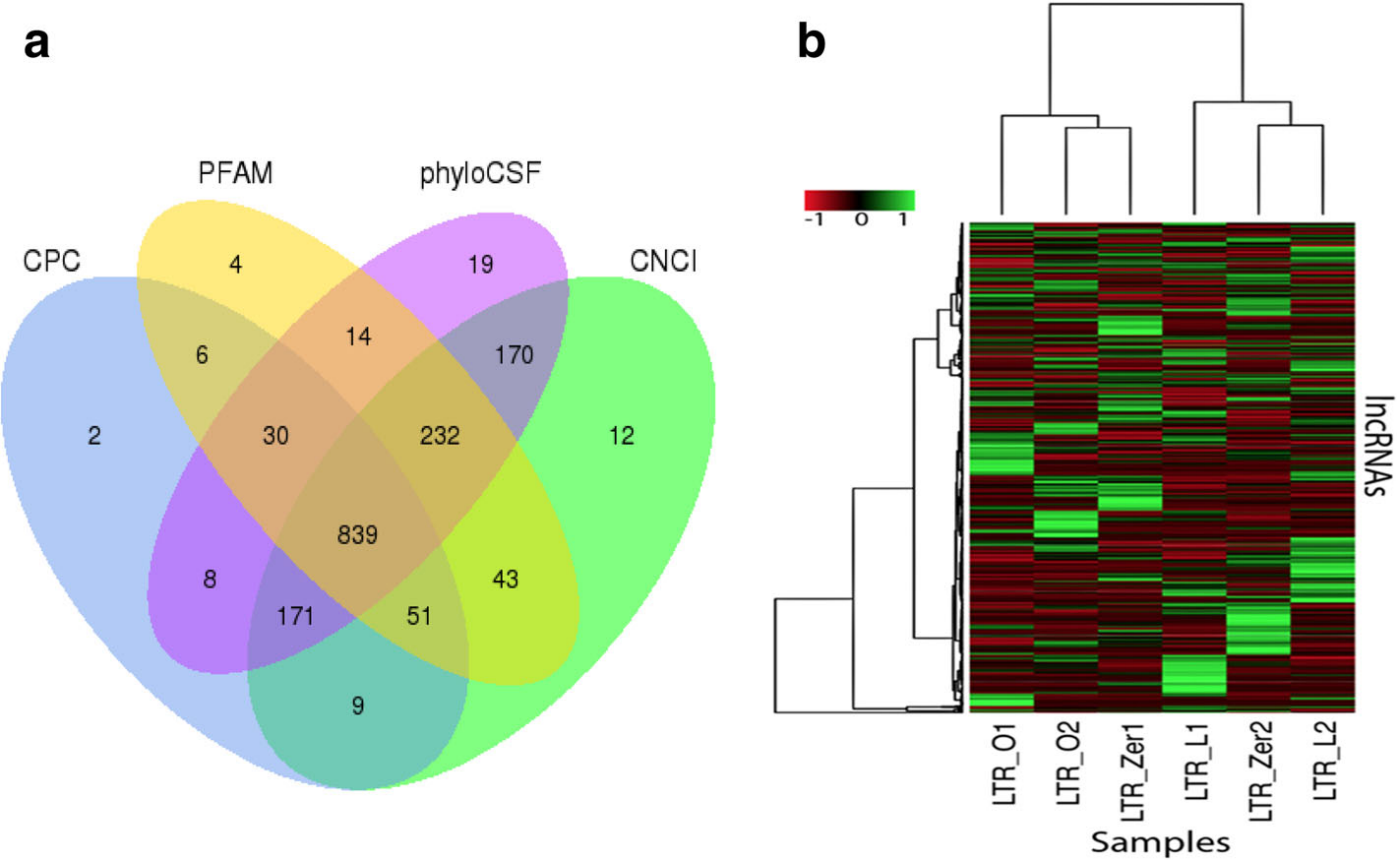
No difference in the lncRNAs among the three experimental conditions. **a** Predicted lncRNAs based on four coding potential filtering methods. CPC, Coding-Potential Calculator; PFAM, Protein FAMily analysis; PhyloCSF, Phylogenetic Codon Substitution Frequency; CNCI, Coding-Non-Coding Index. **b** Expression level distribution of the 839 lncRNAs in the six samples (FPKM values are z-score normalized)

Altogether, 1615 transcripts were identified as candidate lncRNAs (see Additional file 2: Figures S2 and S4 for details). These candidate lncRNAs were then subjected to four coding potential prediction methods. A total of 839 lncRNAs were predicted by all the methods (Fig. 2a) and were therefore used in all the subsequent analyses.

As shown in Fig. 2b, there was no clear clustering of samples from the same condition: LTR_L2 showed higher similarity to LTR_Zer2 than to LTR_L1, and LTR_O2 showed higher similarity to LTR_Zer1 than to LTR_O1. Among the 839 lncRNAs, 38 were identified to be differentially expressed for the L vs. Zero comparison at a *p*-value < 0.05, but none remained significant for the adjusted *p*-values controlling the false discovery rate (FDR) at 0.10 due to multiple testing. 40 lncRNAs were differentially expressed for the O vs. Zero comparison at *p*-value < 0.05, but only one lncRNA, TERC, remained statistically significant for the adjusted *p*-values; 53 were differentially expressed for the L vs. O comparison, but only two lncRNAs, TERC and SCARNA2, remained significant for the adjusted *p*-values. Interestingly, the lncRNA TERC showed differential expression levels for all pairwise comparisons of the three conditions (albeit not significant for the L vs. Zero comparison at the adjusted *p*-value), with the highest expression level under condition L, > 2-fold increase compared to condition O, and a 1.5-fold increase compared to the control (LTR_Zero). The lncRNA SCARNA2 showed the lowest expression level under condition O, followed by increased expression for the control condition (~ 1.4 fold), and condition L (~ 1.7 fold).

### Differentially expressed mRNAs

Altogether, 142,791 mRNAs were compared for differential expression among groups. With a false discovery rate of 0.10, four genes (DSC3, EGF, TRIM26, FHDC1, see Additional file 1: Table S5) were differentially expressed between the L and Zero samples, 24 genes were differentially expressed between the O and Zero samples (Additional file 1: Table S5), and 63 genes were differentially expressed between the L and O samples (Additional file 1: Table S5). Gene Ontology analysis revealed no statistically significant enrichment of any specific categories (results not shown). Comparison of the genes across these three lists of differentially expressed genes for the three pairwise comparisons showed that only one gene, TRIM26, was more robustly down regulated in the L samples (FPKM = ~ 1.4) than in both the O (FPKM = ~ 4.5) and Zero (FPKM = ~ 3.9) samples (all pairwise comparisons are statistically significant). REPS2 was significantly upregulated in both the O and L samples compared to the Zero control, but only showed a statistical significance in the O vs. Zero sample comparison for the adjusted *p*-value; in the L vs. Zero sample comparison, although the *p*-value was significant, the adjusted *p*-value was not. There were 21 genes differentially expressed in the O samples compared with either the L or Zero samples (but not between the L and Zero samples, Table 2). Interestingly, all these 21 genes were significantly downregulated in the O samples as compared to those in both the L and Zero samples. Also interesting was that one third of these genes were histone related: HIST1H2AB, HIST1H2AD, HIST1H2AM, HIST1H4J, HIST2H2AC, HIST2H2BF, HIST2H3D. This result suggestsed that there were no apparent upregulated changes from Zero to LTR_L in all mRNA transcripts. However, LTR_O significantly downregulated some genes. Since the dCas9-SAM was expected to activate the mRNA expression of any potential off-target genes, these downregulated genes might not be directly related to the action of the dCas9-SAM activation system. However, these downregulated genes were specific for the msgRNA LTR_O, and histone-related genes were the most striking, perhaps implying that LTR_O-mediated LTR transcription activation may exhaust some histone proteins. It was unlikely that LTR_O induced direct suppression of several histone genes, unless the enriched transcriptional activator (VP64, p65, HSF1) by the dCas9-SAM via LTR_O msgRNA might suppress histone genes by interacting with their transcriptional complex. It was also possible that LTR_O affected some genes such as TERC and REPS2 that might negatively regulate the expression of these histone genes.

**Table 2:** 21 genes that are significantly downregulated in the O samples as compared to the Zero and L samples

Genes	LTR_O_FPKM	LTR_Zero_FPKM	log_2_(fold)	LTR_L_FPKM	log_2_(fold)
HNRNPAB	6.55	38.66	− 2.56	43.94	−2.75
PTP4A2	3.62	20.44	−2.50	23.02	−2.67
B4GALT2	2.07	6.02	−1.54	6.07	−1.55
C4orf48	4.99	11.81	−1.24	11.15	−1.16
TPGS1	3.36	7.16	−1.09	8.80	−1.39
HPCAL1	4.01	8.33	−1.05	8.39	−1.06
SLBP	10.55	20.53	−0.96	20.64	−0.97
CITED4	3.56	6.79	−0.93	7.66	−1.11
HIST2H2BF	97.90	175.32	− 0.84	176.52	− 0.85
TMEM160	8.40	14.67	−0.80	16.45	−0.97
HIST2H2AC	444.08	750.59	−0.76	850.05	−0.94
C17orf89	57.82	95.66	−0.73	109.36	−0.92
IER5L	6.61	10.87	−0.72	13.23	−1.00
CEBPD	16.45	26.54	−0.69	29.57	−0.85
HIST2H3D	248.87	400.31	−0.69	425.10	−0.77
HIST1H2AB	336.25	536.04	−0.67	587.46	−0.80
HIST1H2AM	468.32	743.47	−0.67	809.48	−0.79
MIF	128.97	200.45	−0.64	247.69	−0.94
HIST1H4J	1102.07	1656.42	−0.59	1819.34	−0.72
CYBA	179.61	268.40	−0.58	308.07	−0.78
HIST1H2AD	722.53	1057.74	−0.55	1177.36	−0.70

### SNP and indel analysis

**Fig. 3:**
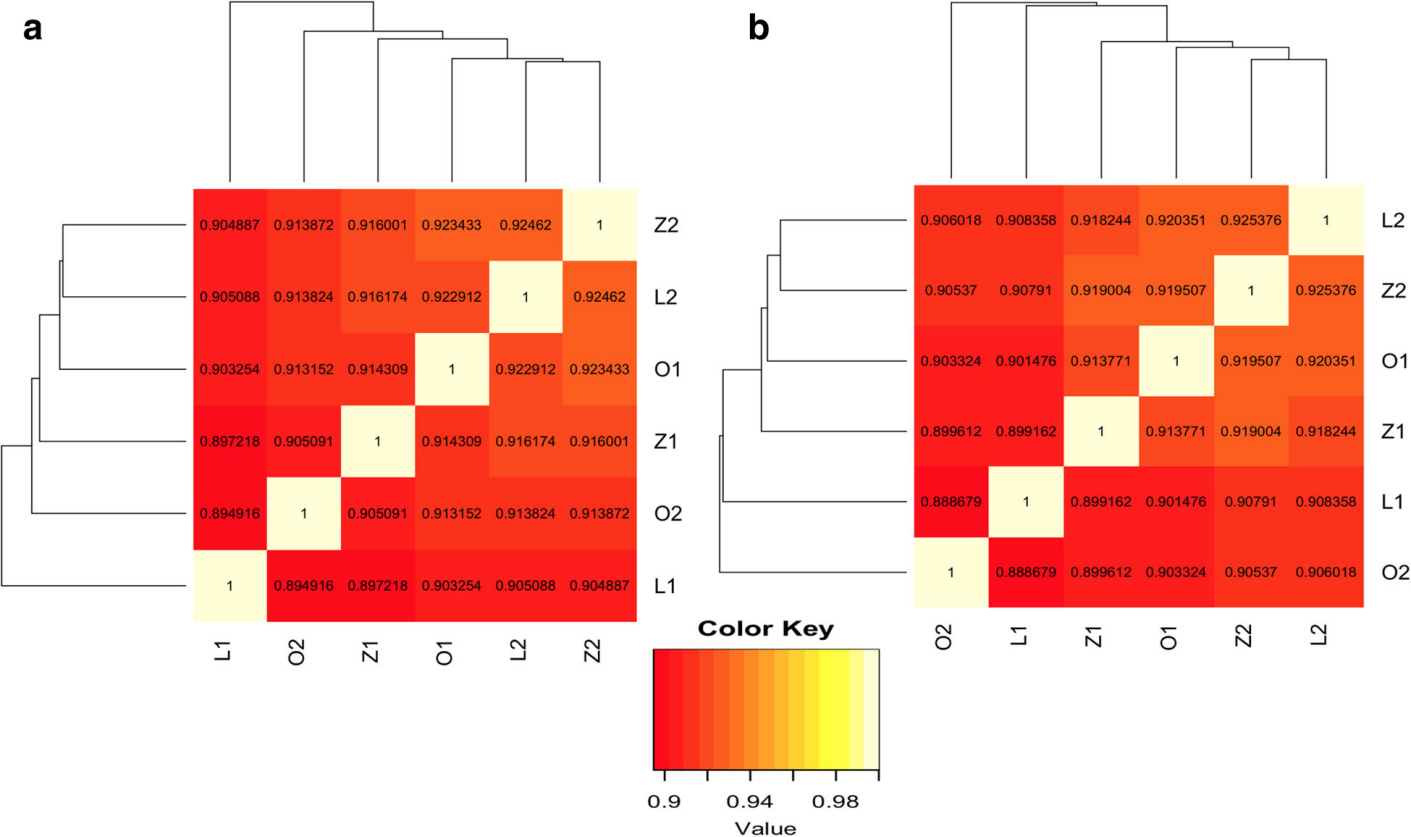
Hierarchical clustering of the six samples based on the Jaccard index for SNPs (**a**) and indels (**b**)

To examine whether the dCas9-SAM epigenome editing had an effect on the rate of genetic mutations, SNPs and indel variants in all the samples were identified using GATK2 [70]. Totally, there were 733,334 SNPs and 36,715 indels identified in the six samples. The Jaccard index was computed for each pair of samples where the number of reads that supported the called SNPs and indels was greater than or equal to 20. Figure 3 showed the Jaccard index matrix and clustering result of the six samples for both SNPs and indels. The Jaccard index was high for all sample comparisons, ranging from 0.895 (O_2_ vs. L_1_) to 0.925 (Z_2_ vs. L_2_) for SNPs, and from 0.889 (O_2_ vs. L_1_) to 0.925 (Z_2_ vs. L_2_) for indels. The clustering result revealed no clear grouping within the same conditions (that is, L samples grouped together, O samples grouped together, or control samples grouped together), suggesting that there were no systematic differences in SNP and indel variations between different editing conditions.

### Very similar distribution of alternative splicing events among the three groups

**Fig. 4:**
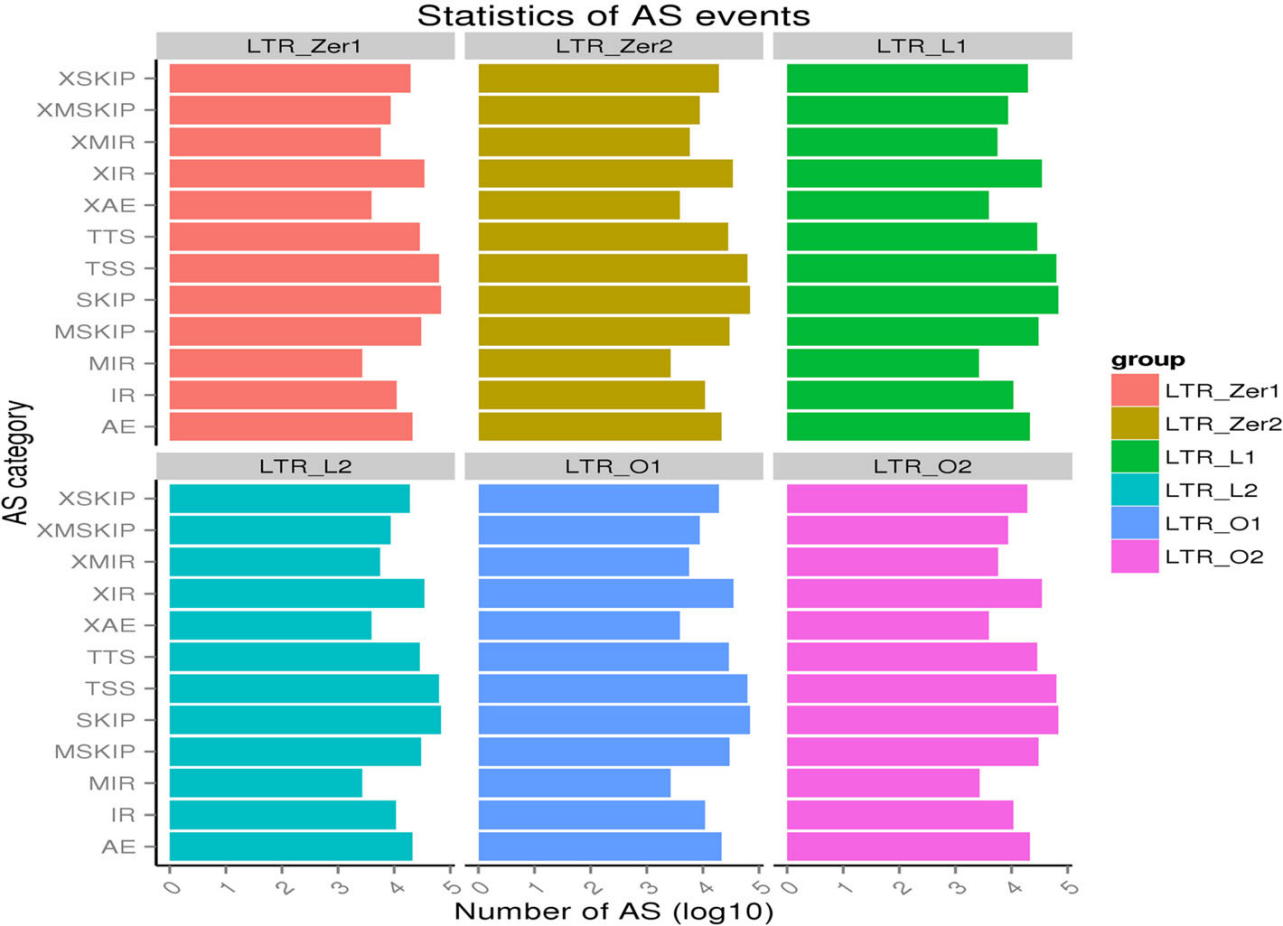
Summary statistics of the 12 types of alternative splicing in the six samples. The number of events for each type is log_10_ transformed

Alternative splicing is an important means for increasing the diversity of transcripts and proteins. In fact, a majority of mammalian genes have around 2~ 12 mRNA isoforms, with some having a few thousand isoforms [76]. Therefore, characterizing the off-target effects of dCas9 epigenome editing is incomplete without considering how alternative splicing might be affected among different groups as compared to the control. To investigate in detail how isoforms or exons might be affected, alternative splicing events were first classified into 12 types as illustrated in Additional file 2: Figure S4 using ASprofile [71]. The number of each type of alternative splicing event for the six samples was shown in Fig. 4 (also see Additional file 1: Table S6). The total number of alternative splicing events ranged from 297,334 to 298,098 with the two LTR_O samples (O1: 298, 098; O2: 297,999) having the highest number of alternative splicing events, followed by LTR_Zer2 (297,789), LTR_L2 (297,763), LTR_Zer1 (297,580), and LTR_L1 (297,334). The distribution of different types of alternative splicing was very similar among the six samples, and there was no significant difference either within or between groups (all the pairwise Chi-square tests’ *p*-values are greater than 0.98).

**Fig. 5:**
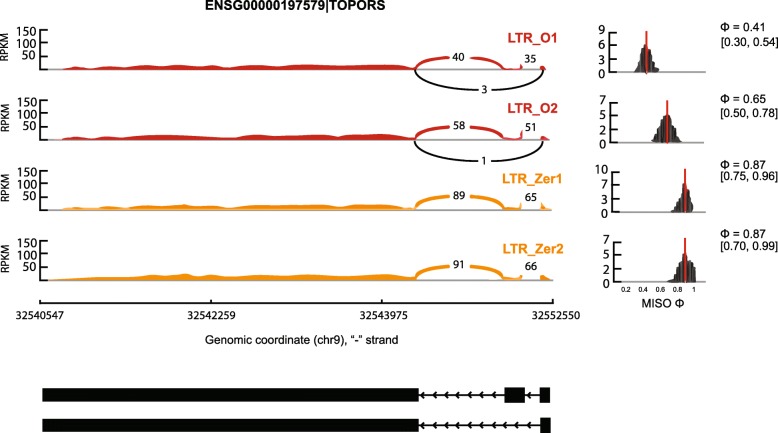
The sashimi plot showing exon skipping in TOPORS that exhibits significant differential regulation between the LTR_O group and the control group. The top left panel shows the FPKM of reads that supports the corresponding exons and exon junctions in the two LTR_O samples and two control samples, respectively. The top right panel shows the posterior distribution of Ψ (the fraction of inclusive isoform), with the red line denoting the estimated Ψ and grey lines the 95% confidence interval of Ψ. The bottom panel shows the two transcripts due to exon skipping in the bottom transcript

To further examine whether isoforms produced by alternative splicing differed in expression level among the three groups, the MISO (mixture-of-isoforms) model [72] was used to determine the isoforms that differentiate the groups. MISO uses a Bayesian statistical model to estimate the expression level of different isoforms/exons and identifies differentially regulated isoforms by the Bayes factor (BF) that calculates the odds of differential regulation of isoforms or exons. Five major types of alternative splicing events, alternative 3′ splice sites (A3SS), alternative 5′ splice sites (A5SS), mutually exclusive exons (MXE), retained intron (RI), and skipped exon (SE), were analyzed and compared among the three groups. Table 3 showed the genes that exhibited significant differential isoform regulation between the group comparisons. Figure 5 showed an example of the TOPORS gene exhibiting significant differential exon skipping in LTR_O samples compared to the Zero samples. Altogether, there were not many differential isoform regulations between the groups. For example, of the 7244 A3SS events compared between the L samples and Zero samples, only seven (< 0.1%) had significant differential isoform regulation. In fact, the percentage of significant differential isoform regulations between groups for the three pairwise comparisons (L vs. Zero, O vs. Zero, L vs. O) ranged from 0.097 to 0.111% for A3SS, from 0.130 to 0.2% for A5SS, from 0.180 to 0.181% for MXE, from 0.122 to 0.197% for RI, and from 0.081 to 0.112% for SE. Taken together, less than 0.2% of the alternative splicing events considered showed differential isoform regulations between the groups, suggesting no genome-wide systematic alternative splicing changes occurred due to the dCas9 editing. Moreover, comparison of the list of genes with differential isoform regulation to the list of differentially expressed genes (Additional file 1: Table S5) showed that only DSC3 had differential exon regulation between the L and Zero samples, and DSC3 was also significantly downregulated in the L samples compared to the Zero samples.

**Table 3:** Comparison of differential isoform regulation between the three groups. The genes in bold font are those shared by two pairwise comparisons. The numbers in parenthesis are the number of events considered for the particular group comparison

AS types	L vs. Zero	O vs. Zero	L vs. O
A3SS (# of events)	(7244)	(7143)	(7239)
	C8orf22	ANKRD11	BMP1
	CLSPN	C11orf48	NFAT5
	COBLL1	CNOT2	**OCEL1**
	DAK	GNB2L1	ORMDL1
	JOSD1	SETMAR	ST5
	**OCEL1**	YWHAB	ZNF84
	PIH1D1	ZNF587	
A5SS (# of events)	(5399)	(5350)	(5407)
	C17orf70	ANGPT1	HILPDA
	MTMR2	CLEC2D	NBPF11
	NOC2L	LAMA4	NDUFV2
	RP5-1198O20.4	NAA60	NT5C
	SMARCC2	SLC50A1	OXLD1
	TWF1	**TYSND1**	RP4-583P15.15
	ZNF30		SRRM1
			TBC1D7
			**TYSND1**
			VPS52
			chr1:32336239:32335947
MXE (# of events)	(4959)	(4946)	(5006)
	**CNOT1**	**CNOT1**	AKIRIN1
	DBNL	EIF4G2	DDHD2
	DPY30	HMGN1	DEK
	MPPE1	PTRH1	DPP3
	PLA2G6	**RPS6KC1**	ELMOD3
	SPDL1	TMBIM4	MPV17L
	TMBIM6	**TMEM116**	**RPS6KC1**
	UQCC1	chr7:143284899:143284974:+@chr7:143285348	TCTN1
	WBP1		**TMEM116**
RI (# of events)	(4109)	(4057)	(4084)
	**CENPV**	**BAX**	**BAX**
	MRRF	CAPRIN2	FANCI
	RP11-5A19.5	CDK5RAP3	HSD17B4
	RPRD2	**CENPV**	MTA1
	SMTN	GPS2	TAB3
		IMPDH2	
		QARS	
		SERAC1	
SE (# of events)	(25,942)	(25,835)	(25,969)
	C2CD5	**AC013394.2**	**AC013394.2**
	CDC42BPA	AGPAT2	AC124789.1
	DCTD	**ATG7**	ARID1B
	DSC3	**B3GALNT2**	ATG10
	GRB10	**BCL2L12**	**ATG7**
	**HMGN1**	CENPU	**B3GALNT2**
	KCTD17	CMTR2	BBS1
	LINC00570	GABPB2	**BCL2L12**
	MIPOL1	**HMGN1**	BTBD7
	**MRPL52**	IMMP1L	CD320
	NCSTN	KDM6A	CD59
	NUMB	KLHL5	DCTD
	PDE4DIP	**MAPK9**	LINC00472
	PXK	**MRPL52**	**MAPK9**
	RAB40B	**PTK2**	MIR4435-1HG
	SCMH1	SETD8	**PTK2**
	SPATA20	SMURF2P1	RHBDD1
	**TMEM139**	**SP3**	RP4-717I23.3
	TTC23	**TBL1XR1**	RPS6KB2
	ZNF138	TINF2	**SP3**
	ZSCAN21	**TMEM139**	ST20-MTHFS
		TMEM189	**TBL1XR1**
		**TOPORS**	**TOPORS**
		TRIP6	UBE2I
		UBE2I	YDJC
		VWA9	ZNF639
		ZNF584	ZNF678
		chr7:143284899:143284974

## Discussion

Determining off-target effects from CRISPR/Cas9-based genome editing in a thorough and highly sensitive manner has been a great challenge in the field [6, 77–79]. Apart from ongoing extensive work in optimizing the technology to minimize off-target cleavage [39, 80–82], serious effort has also been devoted to examining the off-target effects resulting in changes at the levels of genomes and transcriptomes [50, 52, 83–89]. In particular, the specificity of the dCas9-SAM system itself has been validated by mRNA-seq analysis [17, 28, 30], although the dCas9-VP160 alone (in the absence of sgRNA) has been shown to reactivate latent HIV-1 in U1 cells [90]. Here, deep sequencing of transcriptomes of human cells after epigenome (transcriptional) editing by HIV-specific msgRNA/dCas9-SAM was performed, and a comprehensive analysis was done to examine any potential off-target effects of the HIV-targeted msgRNA/dCas9-SAM on the mRNA transcription, lncRNA expression, alternative splicing, as well as genetic mutations including SNPs and indels.

### Off-target effect on the overall mRNA expression level

In terms of mRNA expression, if there were significant off-target effects, many genes would be upregulated in the O and L samples compared to the control group (the genes that are upregulated could differ between the O and L samples), but only a handful of the host genes showed significant difference, most of which were actually downregulated (Additional file 1: Table S5). Specifically, of the 28 genes showing a statistically significant difference, only two, HDGF and REPS2, were significantly upregulated in the O samples compared to the control group. Four genes were found differentially expressed in the L group vs. Zero group comparison, but all of them were downregulated in the L group compared to the Zero group (the control group). It is puzzling that most of the differentially expressed genes were significantly downregulated in the dCas9-SAM editing system (O and L samples) compared to the control group. This phenomenon has not yet been reported anywhere in the literature.

The 12~ 14-bp target sequence near the protospacer-adjacent motif (PAM) region (NGG) is critical for the specificity of Cas9 genome editing [91, 92]. In silico off-target effect prediction for LTR_L and LTR_O was done by blasting > 14-bp target + NGG against the human genome/transcripts as we described previously [20, 21, 23], then comparing the list of potential off-target gene locations with the genes identified in Additional file 1: Table S5. There is no overlap between the two lists, suggesting that genes that show significant expression difference between the two dCas9-SAM edited groups and the control group may not be the direct result of the potential off-target effect.

### Off-target effects on alternative splicing

Comparison with 12 types of alternative splicing events reveals no statistically significant differences between the edited groups (L and O) and the control group (Fig. 4). Moreover, a detailed expression analysis of isoforms caused by five major types of alternative splicing shows only a small number of differential isoform regulations between groups (< 0.2%, Table 3), further suggesting that there are no pronounced genome-wide alternative splicing changes occurring due to the dCas9-SAM editing. DSC3 is the only gene that shows both significant differential exon regulation and expression level differences between the edited group (L) and the control group, but contrary to expectations, is significantly downregulated. Previous studies show about 47~ 74% of alterative splicing events show variation among different human tissues and 10~ 30% of alternative splicing events show variation among individuals [93]. Therefore, comparatively, the level of variation in alternative splicing detected among the three groups (L, O, and control) is 2~ 3 orders of magnitude lower. Although the level of genetic variation among the samples is also lower (less than one order of magnitude, see results on SNPs and indel comparison), these comparisons nonetheless suggest that the off-target effect due to the dCas9 epigenome editing does not include any noticeable changes at the genome-wide alternative splicing level. Since alternative splicing is an important mechanism for increasing transcript and protein diversity [76, 94], and fine-tuning gene expression and function, any off-target effect caused by dCas9 editing could conceivably create undesirable consequences that in turn limit dCas9 usage. The current finding is thus very encouraging for the safe application of dCas9 epigenome editing to reactivate the silent HIVs for their ultimate elimination.

### Off-target effect on lncRNAs

Long noncoding RNA (lncRNA), transcripts longer than 200 nucleotides that cannot be translated into proteins, are derived from 70~ 90% of the mammalian genome while mRNAs are transcribed from only 1% of the genome [95]. These lncRNAs have been shown to play important regulatory roles in chromatin reprogramming and pre- and post-mRNA processing [96–98]. Therefore, any off-target effects on lncRNA expression is also important to consider. Using the pipeline shown in Additional file 2: Figure S2b, 839 lncRNAs (Fig. 2a) were identified in the transcripts and their expression compared in six samples. Results (Fig. 2b) reveal no clear clustering of samples within the same groups and no clear separation among groups. There is no significant lncRNA expression difference between the L group and the control group. Only one lncRNA, TERC, is significantly downregulated in the O samples compared to the control samples. In fact, TERC has the highest expression level under condition L, followed by the control condition, and then condition O. This expression difference does not seem to be directly linked to any off-target effect, as one would expect TERC lncRNA to have higher expressions in both edited groups (O and L groups) compared to the control group. The observation for lncRNA expression is similar to the observation for mRNA expression, because the handful of mRNAs and lncRNAs tend to be downregulated, contrary to an expectation of elevated expressions in the edited groups due to the potential off-target transcriptional activation effect. It is therefore concluded that there is little, if any, detectable off-target effects on lncRNA transcription. As more studies have shown the involvement of lncRNAs in various diseases and cancer [99–102], our current finding is reassuring, and further supports the safe application of dCas9-SAM epigenome editing. Note that the current finding does not preclude the possibility that the off-target effects could upregulate some unknown genetic elements/factors, which in turn suppress/reduce the expression of the mRNA and lncRNAs identified in the current study.

### Off-target effect on SNPs and indels

Off-target-induced mutations are also another important consideration for the safe application of dCas9-SAM system in clinical settings. Although dCas9 itself does not induce indels or SNPs directly due to its lack of endonuclease activity, it is possible that the dCas9-SAM system induces indels indirectly through potential off-target effects on some mutagenic genes. Results (Fig. 3) comparing both SNPs and indels in the six samples did not show any significant off-target effects. Although previous studies have shown that RNA-guided endonuclease mediated genome editing can induce off-target indel mutations [92, 103–106], numerous studies have also shown that off-target mutations can be effectively reduced and possibly eliminated by careful selection of unique target sequences and guide RNA and Cas9 variant optimization [107]. One cautionary note is that since SNPs and indels were identified using RNA-seq data, the current study cannot address whether there is any significant mutagenic effect due to the dCas9 epigenome editing in non-transcribed regions.

## Conclusion

To the authors’ knowledge, this study is the most comprehensive and exhaustive characterization of the off-target effects on transcriptomes after HIV-targeted dCas9-SAM epigenome editing. Analysis of known types of RNAs reveals no significant difference between transcriptomes of HIV-targeted and non-targeted msgRNA-treated human cells, supporting the contention that msgRNA-directed dCas9-based SAM technology can be safely used to reactivate dormant HIV for an effective “shock-and-kill” strategy to finally eliminate the virus [108]. One caveat with the current study is that there were only two replicates for each group, which limits the statistical power of the study. Future work needs to include more replicates. Additionally, further assessment of the potential off-target effects with the dCas9-SAM system in human primary cells and preclinical animal models is warranted.

## Additional files

Additional file 1: **Table S1.** Statistics of RNA-Seq quality reads. **Table S2.** Mapping results. **Table S3.** Validation of dCas9-SAM mRNA and sgRNA expression (transcripts per million). **Table S4.** Distribution of reads in known types of RNAs. **Table S5.** Differentially expressed mRNA transcripts for all the three pairwise comparisons of the samples (O vs Zero, L vs Zero, and O vs L). **Table S6.** Distribution of the 12 types of alternative splicing events across samples. (XLSX 30 kb)

Additional file 2: **Figure S1.** Distributions of the mapped reads in the genome for the six samples. **Figure S2.** Workflow charts for RNA-seq analysis. (a) Library construction. (b) lncRNA filtering by four pipelines to predict candidate lncRNAs based on their structures and noncoding features. **Figure S3**. Statistics of lncRNA filtering. Horizontal axis represents the filtering step and vertical axis represents the number of remaining transcripts after the filtering step. **Figure S4.** Illustration of 12 types of alternative splicing events analyzed by ASprofile (Picture taken from Florea L, Song L, Salzberg SL: Thousands of exon skipping events differentiate among splicing patterns in sixteen human tissues. *F1000Res* 2013, 2:188). (PDF 2000 kb)

## Abbreviations

A3SS: Alternative 3′ splice sites
AS: Alternative splicing
BF: Bayes factor
dCas9: dead CRISPR-associated protein 9
FDR: False discovery rate
FPKM: Fragments per kilobase of transcript per million mapped reads
lncRNA: long noncoding RNA
LTR: Long terminal repeat
MISO: Mixture of isoforms
msgRNA: MS2-mediated single guide RNA
MXE: Mutually exclusive exons
PAM: Protospacer-adjacent motif
RI: Retained intron
SAM: Synergistic activation mediator
SE: Skipped exon
SNP: Single-nucleotide polymorphism
