# Supplementary material for: Comprehensive off-target analysis of dCas9-SAM-mediated HIV reactivation via long noncoding RNA and mRNA profiling
Source: BMC Med Genomics. 2018 Sep 10;11:78. doi: 10.1186/s12920-018-0394-2 (PMC6131778; doi:10.1186/s12920-018-0394-2)
Supplement: Supplementary file 2 — Figure S1. Distributions of the mapped reads in the genome for the six samples. Figure S2. Workflow charts for RNA-seq analysis. (a) Library construction. (b) lncRNA filtering by four pipelines to predict candidate lncRNAs based on their structures and noncoding features. Figure S3. Statistics of lncRNA filtering. Horizontal axis represents the filtering step and vertical axis represents the number of remaining transcripts after the filtering step. Figure S4. Illustration of 12 types of alternative splicing events analyzed by ASprofile (Picture taken from Florea L, Song L, Salzberg SL: Thousands of exon skipping events differentiate among splicing patterns in sixteen human tissues. F1000Res 2013, 2:188). (PDF 2000 kb) [file 12920_2018_394_MOESM2_ESM.pdf]

Supplementary Figure S1. Distributions of the mapped reads in the genome for the six samples.

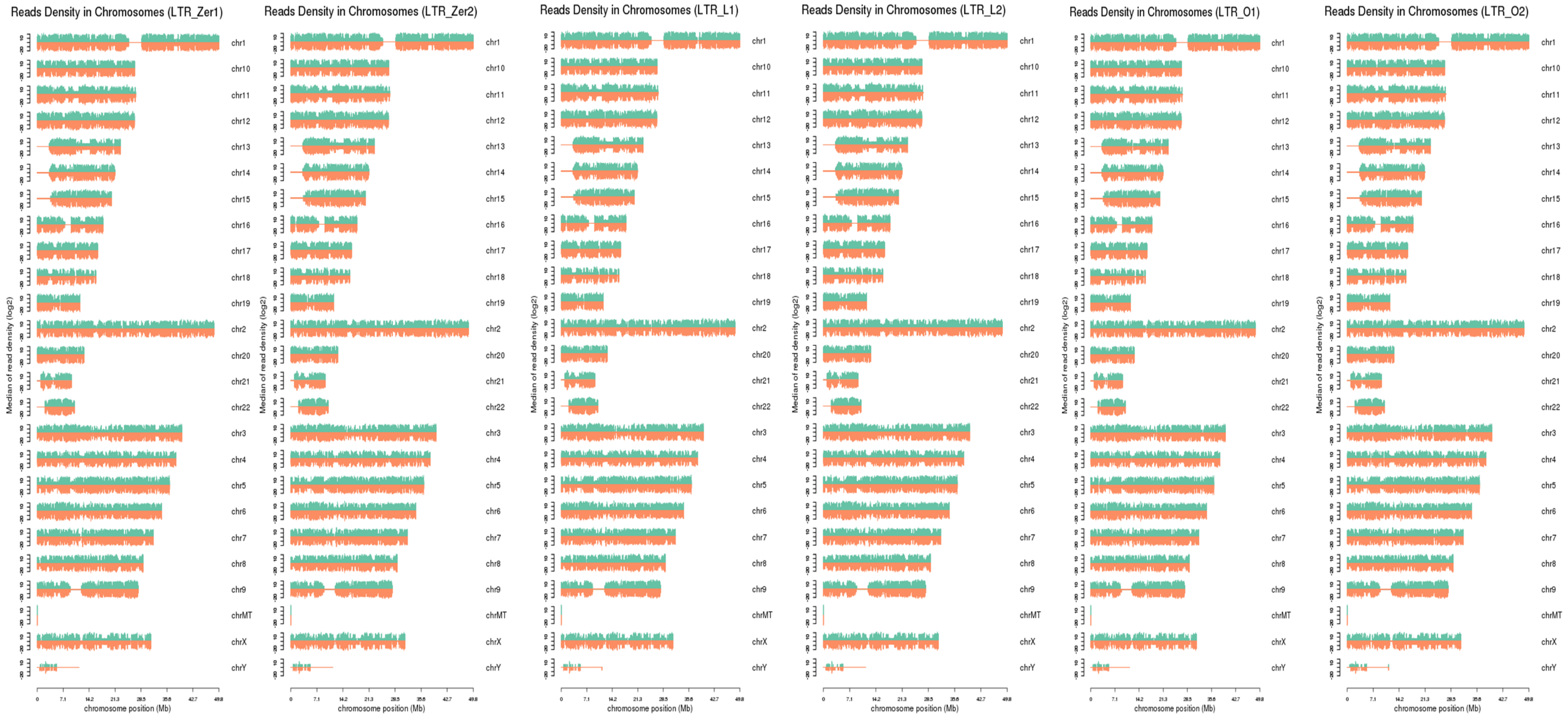

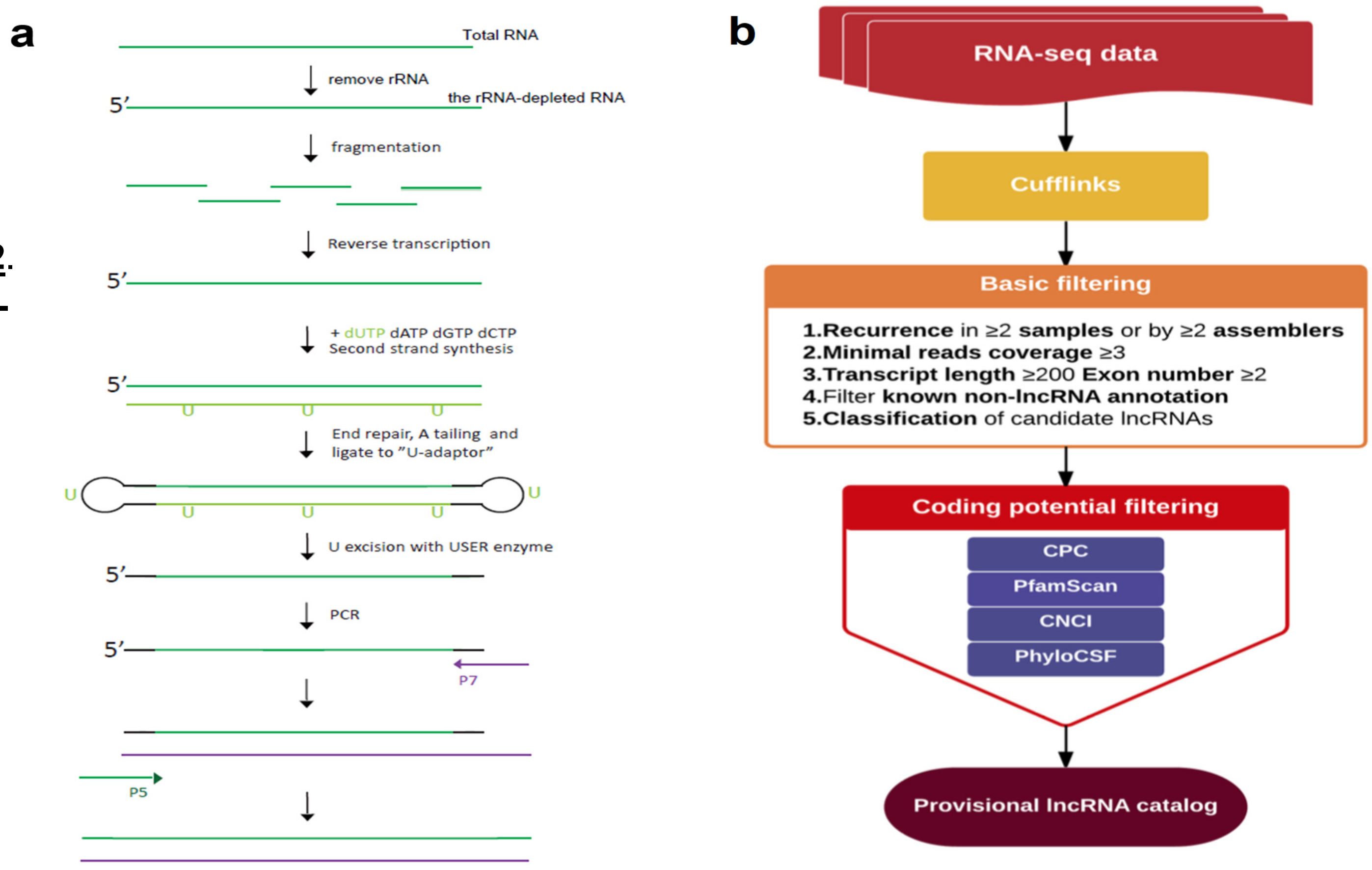

<u>Supplementary Figure S2</u>. Workflow charts for RNA-seq analysis. (a) Library construction. **(b)** lncRNA filtering by four pipelines to predict candidate lncRNAs based on their structures and noncoding features.

<u>Supplementary Figure S3</u>. Statistics of lncRNA filtering. Horizontal axis represents the filtering step and vertical axis represents the number of remaining transcripts after the filtering step.

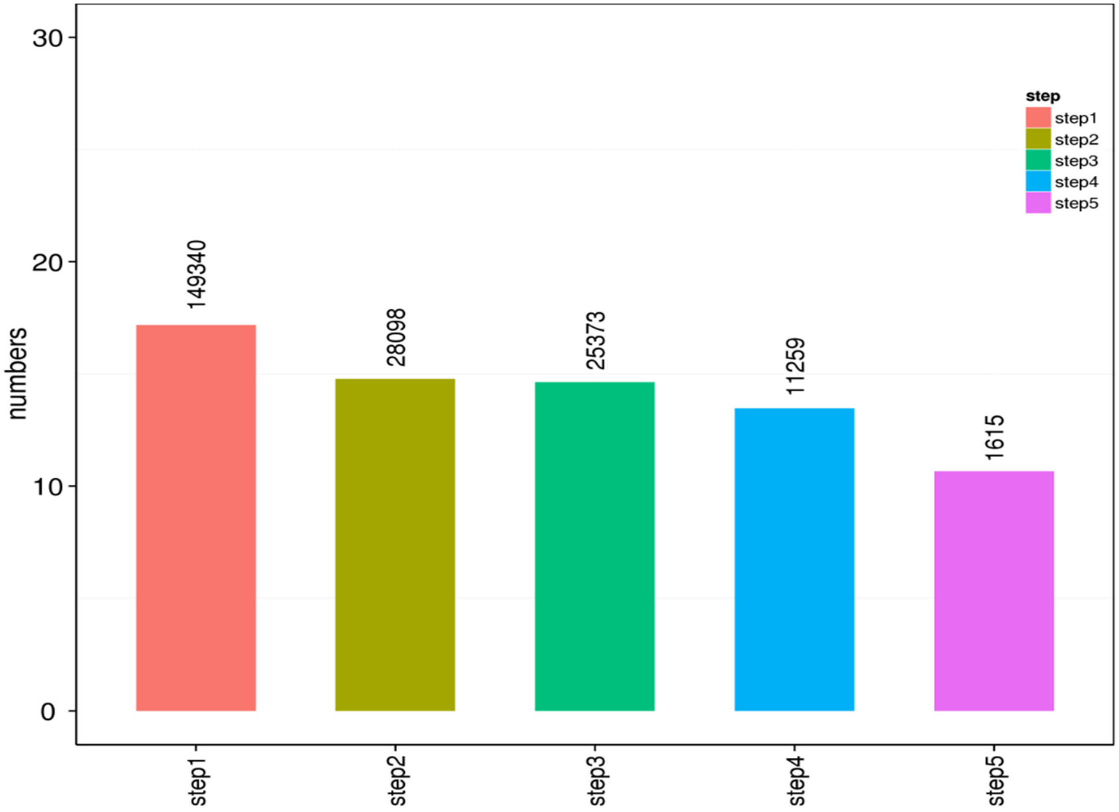

Supplementary Figure S4. Illustration of 12 types of alternative splicing events analyzed by ASprofile (Picture taken from Florea L, Song L, Salzberg SL: Thousands of exon skipping events differentiate among splicing patterns in sixteen human tissues. *F1000Res* 2013, **2:**188)

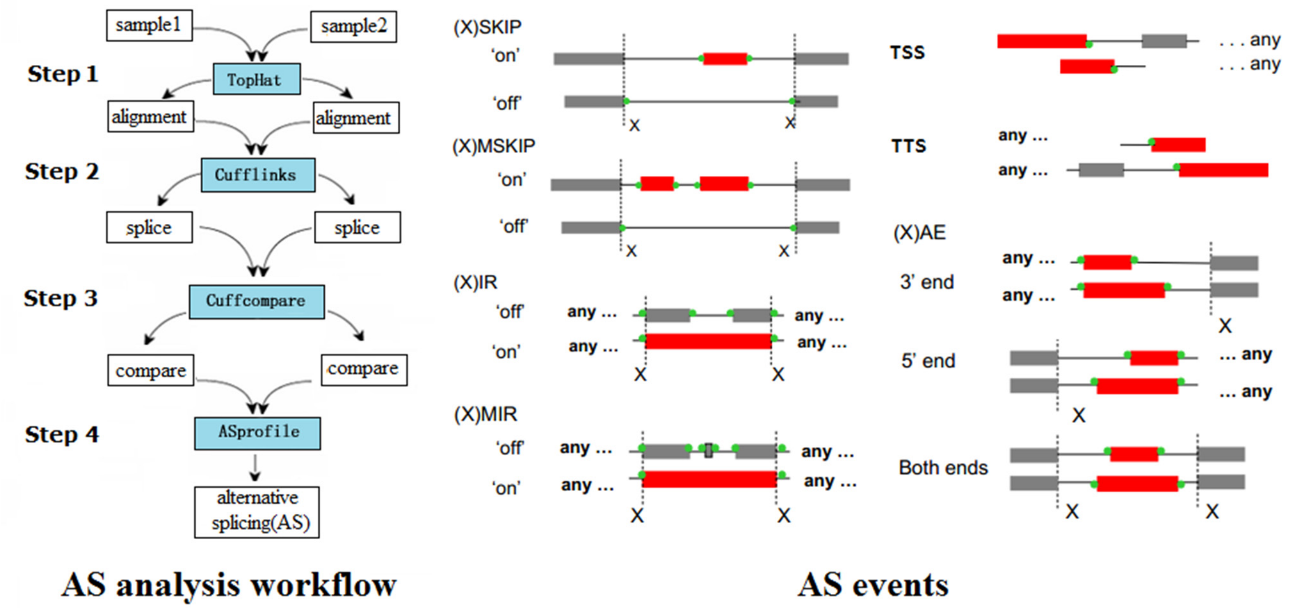

The 12 classes of alternative splicing events:

(1) TSS: Alternative 5' first exon (transcription start site)
(2) TTS: Alternative 3' last exon (transcription terminal site)
(3) SKIP: Skipped exon (SKIP_ON,SKIP_OFF pair)
(4) XSKIP: Approximate SKIP (XSKIP_ON,XSKIP_OFF pair)
(5) MSKIP: Multi-exon SKIP (MSKIP_ON,MSKIP_OFF pair)
(6) XMSKIP: Approximate MSKIP (XMSKIP_ON,XMSKIP_OFF pair)
(7) IR: Intron retention (IR_ON, IR_OFF pair)
(8) XIR: Approximate IR (XIR_ON, XIR_OFF pair)
(9) MIR: Multi-IR (MIR_ON, MIR_OFF pair)
(10) XMIR: Approximate MIR (XMIR_ON, XMIR_OFF pair)
(11) AE: Alternative exon ends (5', 3', or both)
(12) XAE: Approximate AE
